# Supplementary material for: Adequacy of clinical information in X-ray referrals for traumatic ankle injury with reference to the Ottawa Ankle Rules—a retrospective clinical audit
Source: PeerJ. 2020 Oct 8;8:e10152. doi: 10.7717/peerj.10152 (PMC7548068; doi:10.7717/peerj.10152)
Supplement: Supplemental Information 2 [file peerj-08-10152-s002.docx]

| Gender | 1 | male |
| --- | --- | --- |
|  | 2 | female |
| Durationofinjury | 0 | none |
|  | 1 | < 24 hours |
|  | 2 | 1-7 days |
|  | 3 | 7-14 days |
| Clinicalquestion | 1 | fall |
|  | 2 | MVA |
|  | 3 | sporting related injury |
|  | 4 | inversion/eversion injury |
|  | 5 | other |
| Occupationtriagenurseconsultantregistrarmedicalofficersinternsph | 1 | nurse |
|  | 2 | consultant |
|  | 3 | registrar / medical officer / intern |
|  | 4 | physio |
| OARcritNEW | 0 | did not meet crit |
|  | 1 | met crit |
| paininmalleolarzoneNEW | 0 | no |
|  | 1 | yes |
| BonetendernessatposteriordistaltibiaortipofmedialmalleolusNEW | 0 | no |
|  | 1 | yes |
| BonetendernessatposteriordistalfibulaortipoflateralmalleolusNEW | 0 | no |
|  | 1 | yes |
| InabilitytobearweightbothimmediatelyandinemergencydepartmentNEW | 0 | no |
|  | 1 | yes |
| FractureNEW | 0 | no |
|  | 1 | yes |
